# Endoscopic vacuum therapy as a salvage treatment of a life-threatening esophageal perforation

**DOI:** 10.1055/a-2268-5866

**Published:** 2024-03-14

**Authors:** Carlos Robles-Medranda, Domenica Cunto, Maria Egas-Izquierdo, Juan Alcívar-Vásquez, Martha Arevalo-Mora, Jorge Baquerizo-Burgos, Daniela Tabacelia

**Affiliations:** 1 Department of Gastroenterology, Instituto Ecuatoriano de Enfermedades Digestivas (IECED), Guayaquil, Ecuador; 2 20899 Department of Internal Medicine, Larkin Community Hospital Inc, South Miami, United States; 3 434252 Elias Emergency University Hospital, Bucharest, Romania; 4 University of Medicine and Pharmacy, Carol Davila Faculty of Medicine, Bucharest, Romania

The mortality rate of esophageal perforations is 31% 1 . In severe cases, surgery becomes its mainstay management. But what happens when they fail to resolve a life-threatening condition? Endoscopic therapies (stenting, suturing, clipping) become a salvage option showing favorable results 2 ; sometimes, though, they are not enough and require exploration of alternatives 3 4 . We present challenging cases successfully treated with rescue endoscopic vacuum therapy (EVT) ( Video 1 ).

Ex vivo demonstration of endoscopic vacuum therapy technique and application of this procedure as a rescue therapy in life-threatening situations.Video 1

A 19-year-old woman required hemostatic clips and a 23 × 115-mm partially covered self-expandable metal stent (SEMS) for distal esophageal mucosal injury closure after repeat peroral endoscopic myotomy. Empyema and mediastinitis refractory to medical treatment developed and chest tube drainage was performed. Eight days after Ivor-Lewis partial esophagectomy ( Fig. 1 ), hypoxemia and hemodynamic instability arose, and hydropneumothorax and esophageal contrast leakages were found. Pulmonary collections were percutaneously drained via interventional radiology. EVT treated the 2.5 × 3-cm cavity found by esophagogastroduodenoscopy (EGD). During EVT, a 16 F nasogastric tube (NGT) fixed to a sponge (V.A.C. Granufoam Silver Dressing; Acelity, San Antonio, Texas, USA) was positioned intraluminally perorally with an overtube (Guardus overtube; Steris, Mentor, Ohio, USA) and rat tooth forceps. Subsequently, a 14 F NGT was intranasally fixed distal to the 16 F NGT and proximal to the vacuum device placed at –125 mmHg. EVT was repeated seven times every five days ( Fig. 2 ). After computed tomography scan and EGD confirmed leakage closure, the patient was discharged. In the 18-month follow-up, the patient remained asymptomatic, but required stenotic ring dilation.

**Fig. 1:**
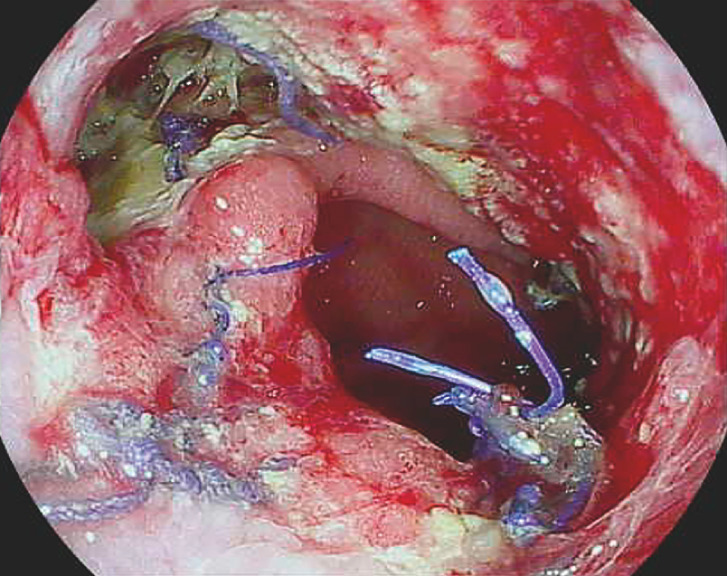
Case 1: Anastomotic dehiscence after Ivor-Lewis esophagectomy.

**Fig. 2:**
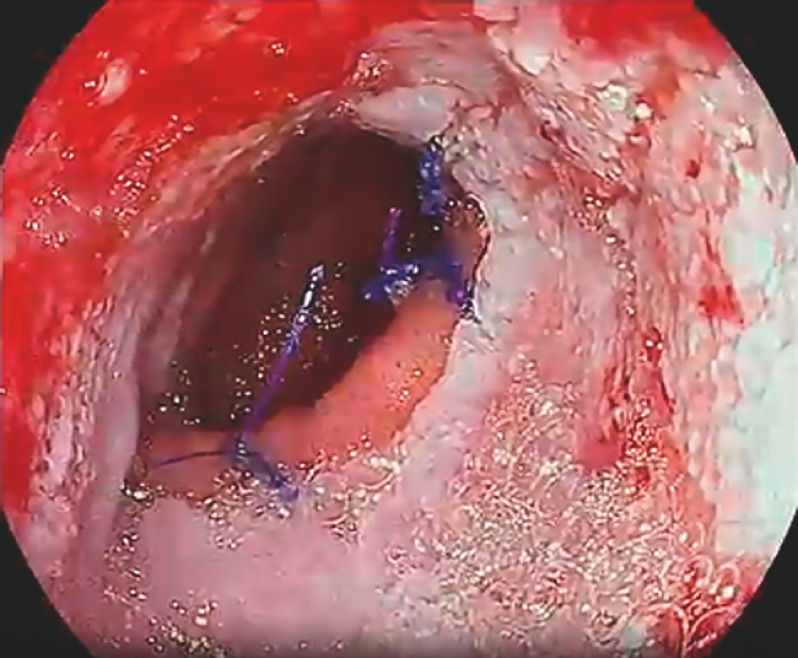
Case 1: Granulation tissue revealed on follow-up esophagogastroduodenoscopy.

A 75-year-old woman underwent endoscopic ultrasound, confirming a 90-mm pseudocyst. During placement of a lumen-apposing metal stent, cystic bleeding occurred requiring closure with over-the-scope clips. On scope retrieval, a cervical-esophageal tear was seen, needing an 18 × 103-mm SEMS. Acute respiratory distress arose 3 days later, requiring mechanical ventilation. The SEMS was removed and oral contrast leakage confirmed suspicion of esophageal perforation ( Fig. 3 ). EVT was performed placing the endo sponge intraluminally with a polypectomy snare (CoinTip snare, Steris). This procedure was performed two times in total, visualizing granulation tissue after session one. ( Fig. 4 ). On day 9, complete perforation closure and a normal esophagogram were documented ( Fig. 5 ) **.** In the 2-month follow-up, the patient remained asymptomatic.

**Fig. 3:**
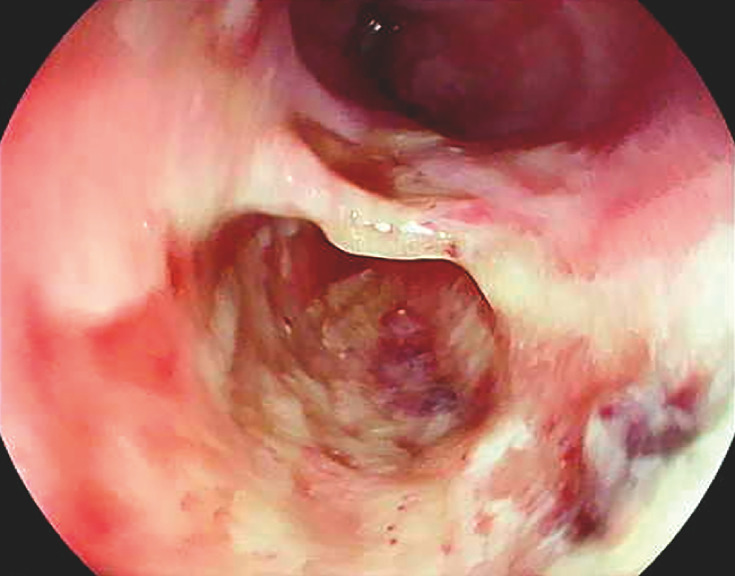
Case 2: Esophageal perforation diagnosed during videoendoscopy.

**Fig. 4:**
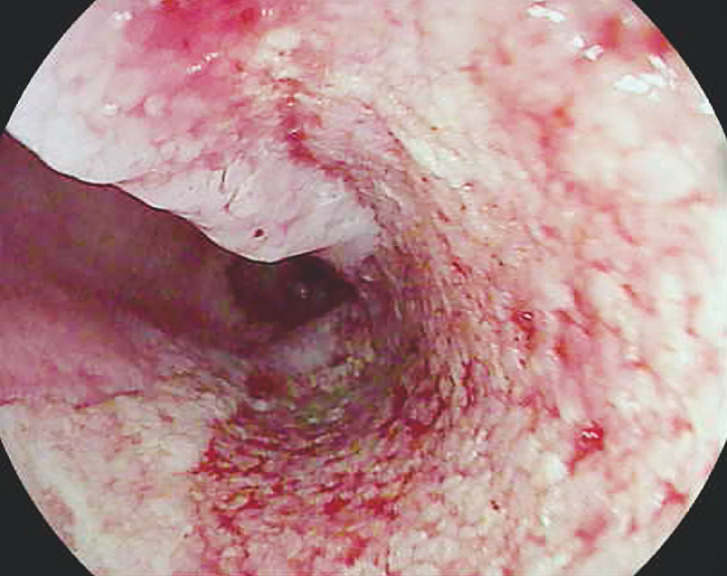
Case 2: Granulation tissue and closure over previous esophageal perforation.

**Fig. 5:**
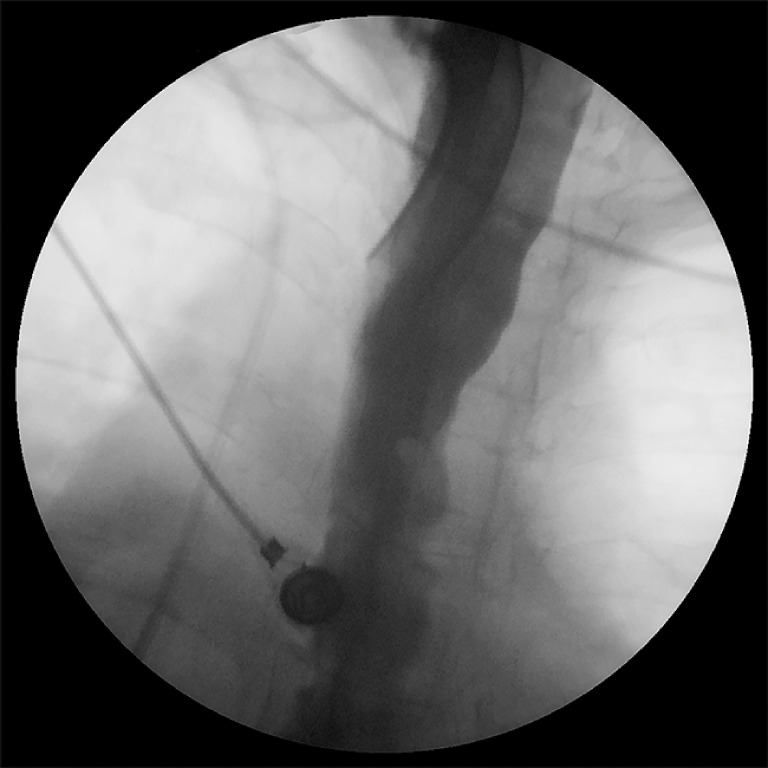
Case 2: Normal esophagogram after endoscopic vacuum therapy.

Endoscopy_UCTN_Code_CPL_1AH_2AG

## Notes

Endoscopy E-Videos https://eref.thieme.de/e-videos
*E-Videos* is an open access online section of the journal *Endoscopy* , reporting on interesting cases and new techniques in gastroenterological endoscopy. All papers include a high-quality video and are published with a Creative Commons CC-BY license. Endoscopy E-Videos qualify for HINARI discounts and waivers and eligibility is automatically checked during the submission process. We grant 100% waivers to articles whose corresponding authors are based in Group A countries and 50% waivers to those who are based in Group B countries as classified by Research4Life (see: https://www.research4life.org/access/eligibility/ ). This section has its own submission website at https://mc.manuscriptcentral.com/e-videos .
